# RBP7 is a clinically prognostic biomarker and linked to tumor invasion and EMT in colon cancer

**DOI:** 10.7150/jca.35180

**Published:** 2019-08-27

**Authors:** Manal Elmasry, Lydia Brandl, Jutta Engel, Andreas Jung, Thomas Kirchner, David Horst

**Affiliations:** 1 Institute of Pathology, Ludwig-Maximilians-Universität München, Munich, Germany; 2 Department of Pathology, Faculty of Medicine, Mansoura University, Mansoura, Egypt; 3 Munich Cancer Registry (MCR), Department of Medical Information Processing, Biometry and Epidemiology (IBE), Ludwig-Maximilians-Universität München, Munich, Germany; 4 German Cancer Consortium (DKTK), Heidelberg, Germany; 5 German Cancer Research Center (DKFZ), Heidelberg, Germany; 6 Institute of Pathology, Charité - Universitätsmedizin Berlin, Berlin, Germany

**Keywords:** RBP7, colon cancer, EMT, invasion, prognosis

## Abstract

RBP7 is a member of the cellular retinol-binding protein (CRBP) family and previous data suggested a link between CRBPs and the malignant transformation of colon cancer cells. Here, we investigated the potential of RBP7 as a predictive biomarker for patients with colon cancer and determined its functional relevance for tumor progression. We analyzed RBP7 protein and mRNA expression in independent tissue collections of colon cancers with recorded follow-up data, including data from TCGA. We used gene set enrichment analyses to characterize its functional role. Effects of RBP7 on migration and invasion were determined in transwell assays. High expression of RBP7 was an independent biomarker of poor cancer specific survival in early and late stage colon cancer, and linked to colon cancer progression. Gene set enrichment analysis revealed a strong association of RBP7, colon cancer invasion and epithelial mesenchymal transition (EMT). Ectopic expression of RBP7 increased migration and invasion of colon cancer cells. Our findings demonstrate that RBP7 is a strong prognostic biomarker in colon cancer that functionally contributes to the malignant phenotype of colon cancer cells. This may aid in risk stratification for the therapeutic management of patients with colorectal cancer.

## Introduction

Colon cancer is one of the most common malignancies, with incidence rates ranking second in women and third in men among all diagnosed cancers. Moreover, it is the third and fourth leading cause of cancer-related deaths in women and men, respectively 1. The prognosis of colon cancer mainly depends on tumor stage. While patients with early and localized disease may be cured by surgical tumor resection, late stage metastasized disease often is fatal 2, 3. However, even patients with early stage colon cancer may show disease relapse and cancer progression despite initial surgical management 4. This indicates the need for prognostic biomarkers that aid in risk stratification of colon cancer beyond clinical staging.

Most colon cancers derive from normal colonic mucosa through mutations in *APC, RAS* and *TP53* genes that transform colonic epithelial cells into invasively growing tumors 5. Deregulation of WNT, MAPK and p53 signaling by these mutations leads to an activation of transcription factors of the ZEB and SNAIL family, which cause loss of epithelial and gain of mesenchymal traits in colon cancer cells 6. This epithelial to mesenchymal transition (EMT) then allows for a destructive infiltration of surrounding tissues, and is considered hallmark of cancer progression 7, 8. Factors that are linked to EMT and invasion in colon cancer may thus be promising indicators of tumor progression.

Cellular retinol binding protein7 (RBP7) contains 134 amino acids and is encoded by the *RBP7* gene, located on human chromosome 1p36.22 9. It is a member of the cellular retinol-binding protein (CRBP) family, which is required for vitamin A stability and metabolism 10. Vitamin A (retinol) and its metabolic products are involved in many biological processes, including epithelial cell proliferation, differentiation, and apoptosis 11. Recent studies demonstrated that some CRBP members and retinol signaling might be relevant for colon cancer progression and cancer stem cell traits 12, 13. Moreover, a significant increase in RBP7 expression has been observed in renal cell carcinomas and thus suggested that it may be associated with the development of certain types of cancer 14. However, in colon cancer, the expression and function of RBP7 yet remains unknown.

In this study, we explored the expression of RBP7 in colon cancer, analyzed associations with clinicopathological characteristics, and determined its functional relevance for tumor progression. We demonstrate that RBP7 may be a useful biomarker that links retinol metabolism to invasive growth, EMT, and poor prognosis in colorectal cancer.

## Materials and Methods

### Clinical samples

Formalin-fixed, paraffin-embedded (FFPE) tissue blocks of colorectal cancer specimens were retrieved from the archives of the Institute of Pathology of the Ludwig-Maximilians-Universität München (LMU). These specimens were obtained from patients who underwent intentionally curative surgical resection between 1994 and 2007. Follow-up data of patients were recorded by the Munich Cancer Registry. Patient identifying information was removed from specimens and data, and the need for consent was waived by the institutional ethics committee of the Medical Faculty of the LMU. Patients with localized colorectal adenocarcinomas and absence of nodal (N0) or distant metastasis (M0) at the time of diagnosis (UICC stage I and II 15), and with no history of receiving adjuvant therapies were included in the study. The information on tumor stage and grade of tumor differentiation was reviewed for all cases. Tissue microarrays (TMAs) of colorectal cancer tissues were constructed with representative 1 mm cores from FFPE blocks, including tumor edges and tumor centers of each case. A total of 219 colorectal cancer cases were available for evaluation. During the follow-up period, patients had died from colorectal cancer in 42 cases (19%).

### Immunohistochemical staining, assessment and scoring

5 µm TMA sections were cut, deparaffinized and stained with rabbit anti-RBP7 polyclonal antibody (Sigma, HPA034749, 1:100 dilution) on a Ventana Benchmark XT autostainer with ultraView Universal DAB detection kits (Ventana Medical Systems). For semi-quantitative scoring, each case then was categorized into barely detectable, weak, moderate or strong expression, based on the extent of positive staining. For quantitative scoring, slides were scanned using a Panoramic Desk digital slide scanner (3D Histech), and analyzed using the QuPath digital image analysis software 16. All scanned images of immunohistochemically stained TMA sections were imported into QuPath to be dearrayed, and computational color deconvolution was applied to separate haematoxylin and 3,3'-diaminobenzidine (DAB) stains, as previously described 17. An automated detection algorithm was used in QuPath to differentiate tumor and non-tumor cells. After calibration of RBP7 immunopositivity thresholds, H-scores were calculated based on the extent and intensity of RBP7 nuclear staining by adding 3x % of strongly stained tumor cells, 2x % of moderately stained tumor cells, and 1x % of weakly stained tumor cells 16. For regional differences, H-scores of RBP7 staining were separately determined in tumor cells at the tumor stroma interface (tumor edge), and 100 µm or more away from the tumor stroma interface (tumor center), for each case. All analyses were conducted in a blinded fashion from clinical outcome.

### TCGA colon cancer data, gene set enrichment analyses (GSEA) and heat maps

Gene expression data (RNA-Seq) and corresponding clinical and mutational data of 457 colon cancer samples were downloaded from TCGA (https://cancergenome.nih.gov/) and cBioPortal (https://www.cbioportal.org/). A ranked gene list was generated by calculating Pearson correlations of RBP7 expression and the expression of 20,531 genes within the TCGA dataset. Then, correlations between this ranked gene list and curated gene sets from the Molecular Signatures Database v5.0 18 were searched for by gene set enrichment analysis (GSEA) 19. The default parameters of GSEA using gene lists of 15 to 500 genes were applied, and analyses were run with 1,000 permutations. Heat maps and clustering for *RBP7* mRNA expression and individual EMT regulators were generated by using Morpheus software (Broad Institute, https://software.broadinstitute.org/morpheus/). For *RAS* and *BRAF* status, activating mutations in codons 12, 13, 61, 117, and 146 of *KRAS* and *NRAS*, and in codon 600 of *BRAF* were considered, respectively.

### Cell culture and ectopic RBP7 expression

HCT116 and SW1222 colon cancer cell lines were obtained from the American Type Culture Collection and cultured in Dulbecco's modified Eagle's medium (DMEM) supplemented with 10 % fetal bovine serum (FBS), 100 U/ml penicillin, and 0.1 mg/ml streptomycin (Biochrom). Cell cultures were maintained in a humidified incubator with 5% CO2 at 37 °C. For ectopic RBP7 expression, a synthetic sequence containing *RBP7* mRNA transcript variant 1 (accession number NM_052960.2) was synthesized through IDT DNA (Coralville, USA) and inserted between BamH1 and Xba1 sites of the pcDNA3.1 mammalian expression vector (Invitrogen), resulting in pcDNA3.1-RBP7. Restriction analysis and Sanger sequencing verified correct insertion. Then, empty pcDNA3.1 or pcDNA3.1-RBP7 were transfected into HCT116 or SW1222 colon cancer cells using Fugene 6 reagent (Promega) according to the manufacturer's protocol. 48 h after transfection, cells were used for subsequent experiments.

### Western blot analysis

Transfected HCT116 and SW1222 colon cancer cells were lysed in RIPA Buffer supplemented with protease and phosphatase inhibitors (Roche). Protein concentrations were measured using DC protein assay kits (Bio-Rad). Protein samples were separated by 10% acrylamide SDS-PAGE and then transferred to PVDF membranes (Merck Millipore). Membranes were blocked in 5% nonfat dry milk for 1 hour, and incubated with rabbit anti-RBP7 polyclonal antibody (Sigma, HPA034749, 1:250) or mouse anti-Tubulin monoclonal antibody (Sigma, 1:50,000) at 4°C overnight, and then treated with HRP‐conjugated anti-rabbit (Sigma, 1:5,000) or anti-mouse (Promega, 1:30,000) secondary antibody at room temperature for 1 hour. A chemiluminescent HRP substrate (Millipore) was applied to detect protein bands using the Li-COR Odyssey Fc imaging system.

### Cell Migration and invasion assay

For migration and invasion assays, 1x10^5^ transfected HCT116 and SW1222 colon cancer cells in 250 μl of serum free medium were seeded into the upper chamber of ThinCert cell culture inserts (8 μm pore size, Greiner Bio-One). For cell invasion assays, the inserts were coated with 100 µl of 1 mg/ml growth factor depleted Matrigel (Corning) before adding the transfected colon cancer cells. 500 µl serum-free medium was added to the lower chamber, and replaced 24 hours later by DMEM containing 10 % FBS. After incubation for 48 hours for migration and 96 hours for invasion, inserts were removed, cells were fixed in 4 % paraformaldehyde and methanol, and stained using 0.1 % crystal violet. The non-migrated or the non-invaded cells were removed by cotton swabs from the upper surface of the filters, and photomicrographs of migrated or invaded cells were taken. For quantification, staining of cells from culture inserts was dissolved in 250 µl of 30 % acetic acid, and absorbance was measured at 590 nm on a Varioskan instrument (Thermo Scientific).

### Statistical analysis

Optimal cut-off levels for RBP7 protein expression after immunohistochemistry and *RBP7* mRNA expression level in TCGA data were determined by receiver operating characteristic (ROC) curve analyses and Youden's index. Cancer-specific survival was measured from the date of tumor resection to the date of death from colon cancer, while deaths of other cause were censored. Survival plots were generated by the Kaplan-Meier method, and groups were compared with the log-rank test. Cox proportional hazards model was used for univariate and multivariate analyses. Cases with missing data were excluded from respective analyses. For invasion and migration, groups were compared by t-tests. Statistics were calculated using SPSS (IBM), and* P* values < 0.05 were considered statistically significant.

## Results

### RBP7 is expressed in colon cancer cell subpopulations

**Figure 1:**
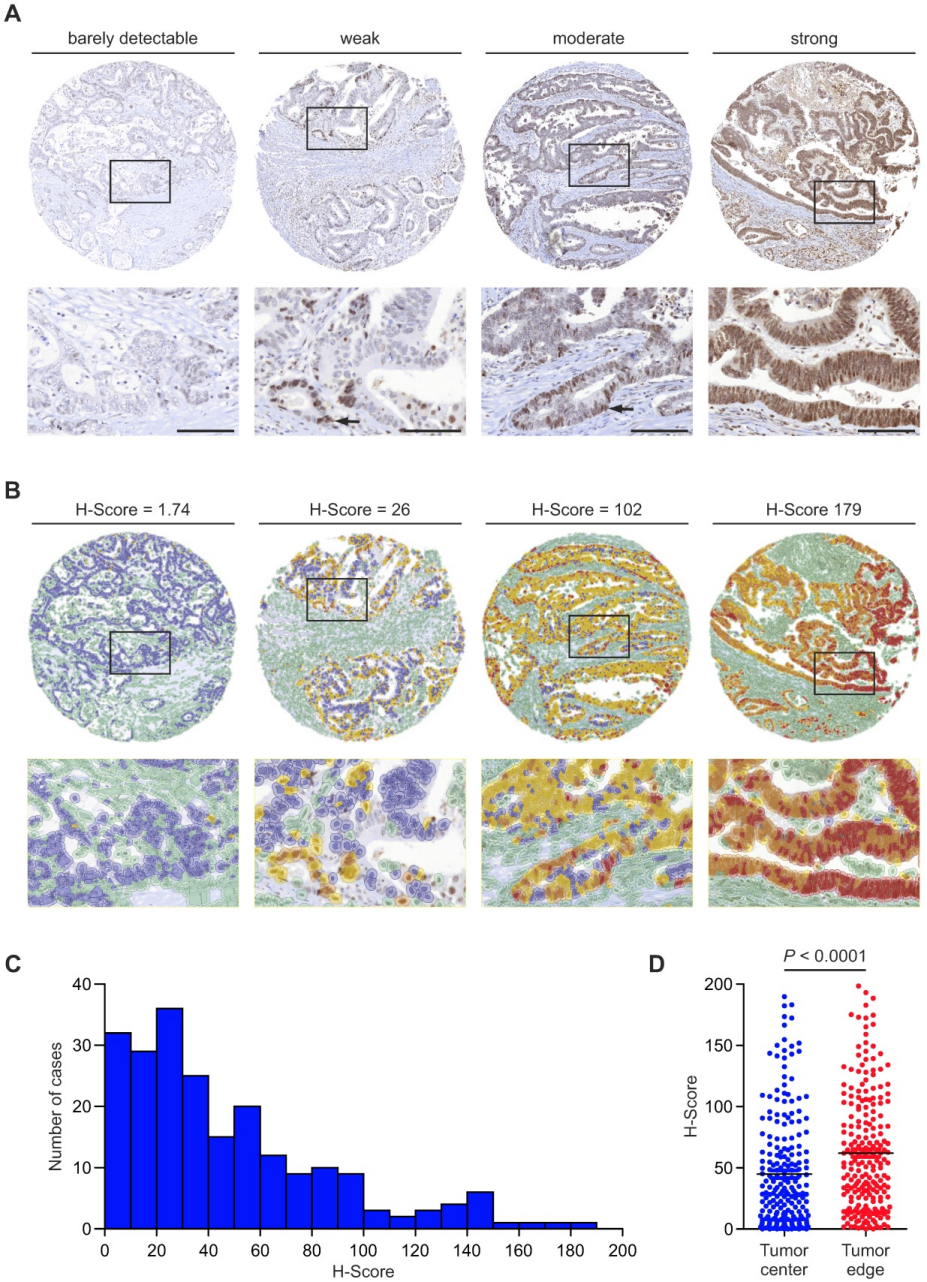
** RBP7 protein expression and distribution in colon cancer. (A)** Detection of RBP7 by immunostaining in primary human colon cancers. Tumors were assigned semi-quantitative categories from barely detectable to strong expression of RBP7. *Arrows* indicate positively stained tumor cells in cases with weak or moderate expression. *Lower panel* images are magnifications of areas boxed in *upper panel* images. *Scale bars*, 100 µm. **(B)** Representative images showing digital quantitative scoring of RBP7 protein expression on the same cases as in (A). Detected cells were color-coded according to their classification. Green, non-tumor cells. Blue, negative tumor cells. Yellow, weakly stained tumor cells. Orange, moderately stained tumor cells. Red, strongly stained tumor cells. H-scores are indicated. *Lower panel* images are magnifications of areas boxed in *upper panel* images. **(C)** Histogram showing the distribution of H-score values in n=219 colon cancer cases. **(D)** Distribution of H-scores, when separately measured in tumor cells at the tumor stroma interface (tumor edge), and 100 µm or more away from the tumor stroma interface (tumor center). Horizontal bars indicate mean and *P* value is t-test result.

To learn about the distribution and expression of RBP7 in colon cancer, we examined a collection of 219 tissue specimens. RBP7 protein was located in the tumor cell nuclei of colon cancers. The number of RBP7 positive tumor cells and expression intensities varied greatly, ranging from barely detectably in few, to strong expression in most tumor cells (Figure 1A). Interestingly, within individual cancers RBP7 expression was not evenly distributed but instead labelled tumor cell subsets, which was most apparent in cases with weak to moderate expression (Figure 1A). Next, in order to assess RBP7 expression objectively, we applied a digital quantitative scoring approach to determine H-scores 16 that integrated the frequency (range 0 % - 100 %) and staining intensity (range 0 - 3) of RBP7 positive tumor cells for each case (Figure 1B). In line with our initial semi-quantitative analysis, H-scores ranged widely among different colon cancers, with a minimum of 0 and a maximum of 184.27 in our case collection (Figure 1B-C). We then analyzed different regions within each tumor, and observed that tumor cells close to the tumor edge showed significantly higher RBP7 expression scores when compared to tumor cells that were located in the tumor center (Figure 1D). These data indicated that RBP7 is expressed in tumor cell nuclei of most colon cancers, increases in expression towards the tumor edge, and can be quantitatively assessed in tumor tissue specimens.

### High RBP7 expression indicates poor outcome in patients with early stage colon cancer

**Figure 2:**
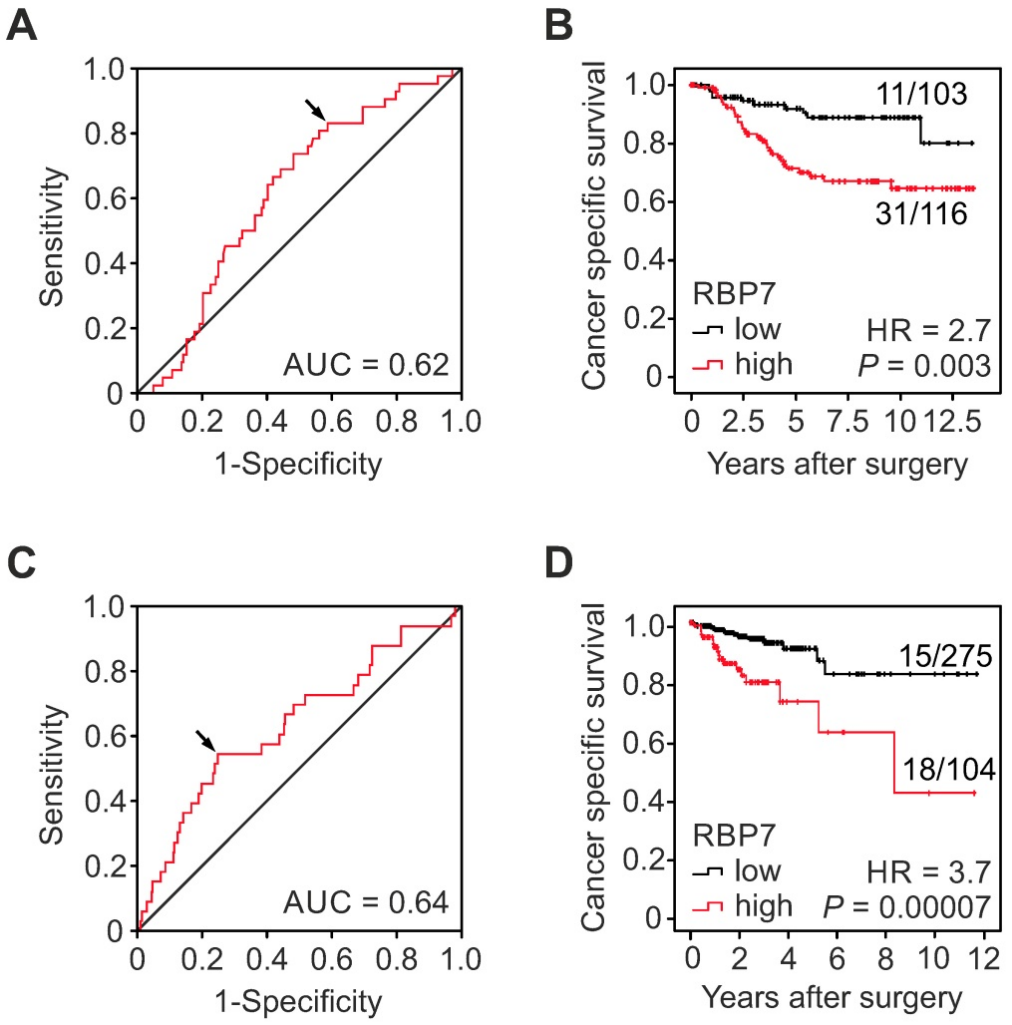
** High RBP7 indicates poor survival in colon cancer patients. (A-B)** Analysis of RBP7 protein expression and cancer specific survival in a case collection of n=219 UICC stage I and II colon cancer cases.** (A)** ROC curve for determining best discrimination thresholds of RBP7 H-scores for tumor specific survival prediction. *Arrow* indicates chosen value for binary classification. AUC, area under curve. **(B)** Kaplan-Meier plot for tumor specific survival of cases with low or high H-scores. *P* value indicates log-rank test result. Ratios on curves indicate the number of events over the number of patients per group. HR, hazard ratio. **(C-D)** Analysis of *RBP7* mRNA expression and cancer specific survival in n=379 colon cancer cases from TCGA **(C)** ROC curve for determining best discrimination thresholds of *RBP7* mRNA reads for survival prediction. Arrow indicates chosen value for binary classification. AUC, area under curve. **(D)** Kaplan-Meier plot for cases with low or high *RBP7* mRNA expression. *P* value indicates log-rank test result. Ratios on curves indicate the number of events over the number of patients per group. HR, hazard ratio.

In order to determine the clinical significance of RBP7 expression in colon cancer, we tested for associations with clinicopathological variables and patient follow-up in our collection of 219 cases, which included UICC stages I and II. Using ROC curve analysis and Youden's index for cancer specific survival, we identified an optimal cut‐off H-score of 32.5 for dichotomal classification into cases with high or low RBP7 expression, respectively (Figure 2A). Indeed, Kaplan-Meier analysis and log-rank testing demonstrated significantly poorer cancer specific survival of patients whose tumors were RBP7 high when compared to RBP7 low cases (*P* = 0.003; Figure 2B). Next, we evaluated correlations of RBP7 high and low expression with other clinicopathological variables by Chi-square testing. High RBP7 expression marginally significantly correlated with high tumor grade (*P* = 0.05), whereas we found no correlations with age, gender, T-category, or UICC-stage (Table 1). Moreover, proportional hazards regression analysis demonstrated that high RBP7 expression was an independent predictor of poor tumor specific survival in this case collection (HR = 2.54; *P* = 0.009; Table 2). These findings suggested that RBP7 is a prognostic marker in early stage colon cancer.

### High *RBP7* expression is an independent predictor of poor survival in colon cancer

For further validation, we next tested for clinical correlations of *RBP7* mRNA levels using publicly available gene expression data of 457 colon cancer cases from TCGA, 379 of which had information on clinical follow-up. ROC curve analysis and Youden's index identified an optimal cutoff score of 21.01 *RBP7* normalized mRNA reads for dichotomal classification of cases (Figure 2C). Also in this data set, Kaplan-Meier analysis and log-rank testing demonstrated a strong positive correlation of high *RBP7* expression and poor cancer specific survival when compared to tumors with low *RBP7* levels (*P* = 0.00007; Figure 2D). We then tested for associations with other core clinical variables, and found that the frequency of high *RBP7* expression increased with increasing T-category, and was higher in tumors that had metastasized to lymph nodes. Other variables including microsatellite instability as well as *RAS* and *BRAF* mutation status were not associated with *RBP7* (Table 3). Furthermore, proportional hazards regression analysis including key clinical variables demonstrated independent prognostic power of high *RBP7* mRNA expression (HR = 2.5, *P* = 0.038; Table 4). Collectively, these data provided additional evidence on the mRNA level that *RBP7* expression is associated with advanced tumor stages and colon cancer progression.

### *RBP7* is linked to invasion and EMT in colon cancer

**Figure 3:**
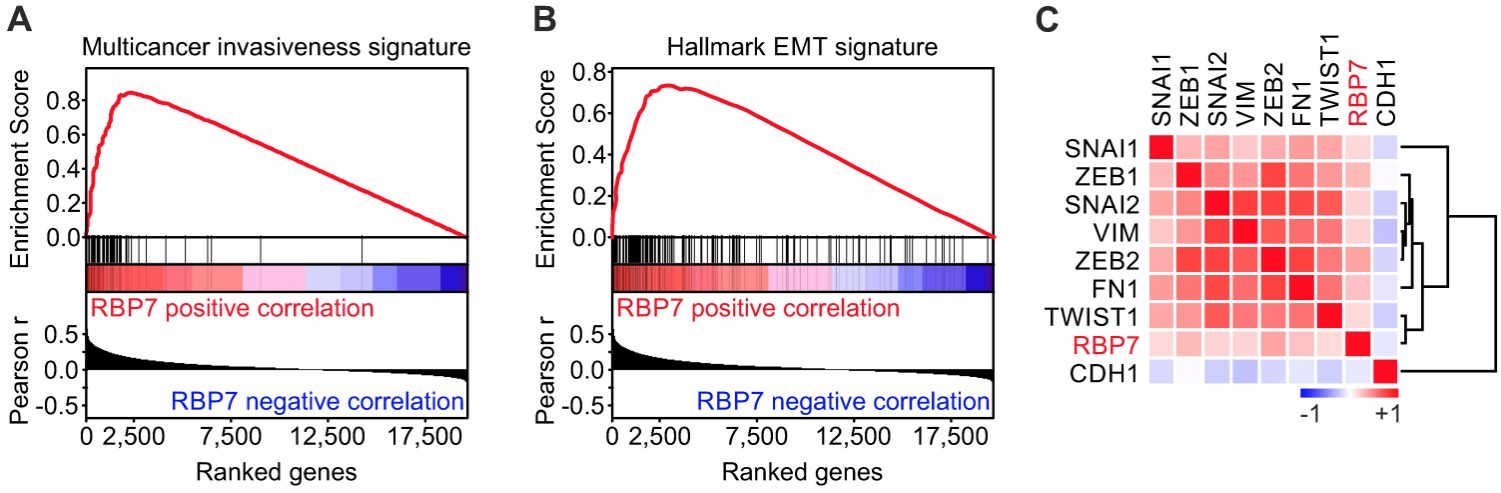
***RBP7* expression is linked to invasion and EMT in colon cancer. (A-B)** Gene Set Enrichment Analyses for genes ranked by Pearson correlation (Pearson r) of expression to RBP7 indicates enrichment for **(A)** multicancer invasion and **(B)** hallmark EMT gene signatures. ES, enrichment score. *P* < 0.001. **(C)** Heat map indicates clustering and positive correlation of *RBP7* expression with colon cancer relevant EMT markers and negative correlation with *CDH1*. Colors indicate Pearson r from -1 (blue) to 1 (red).

To gain insights into the functional role of *RBP7* in colon cancer, we conducted Gene Set Enrichment Analyses (GSEA) using the TCGA dataset. Interestingly, when we tested for associations of *RBP7* expression and curated gene sets (n = 4.762), we found a top enrichment for a multicancer invasiveness gene signature 20, while *RBP7* itself was not part of this gene set (Figure 3A). We then further tested for associations with hallmark gene sets (n = 50) 21, and found the strongest enrichment for genes linked to epithelial mesenchymal transition. Moreover, individual markers that indicate or drive EMT in colon cancer showed a significant overexpression in tumors with high *RBP7* expression, including *ZEB1* (r = 0.27, *P* < 0.0001) and *ZEB2* (r = 0.36, *P* < 0.0001) (Figure 3C). In contrast, the epithelial differentiation marker *CDH1* negatively correlated with *RBP7* (Figure 3C). Importantly, *RBP7* itself again was not part of this EMT gene set. These findings suggested a previously unknown functional link of *RBP7*, invasion and EMT in colon cancer cells.

### Overexpression of RBP7 enhances migration and invasion of colon cancer cells

**Figure 4:**
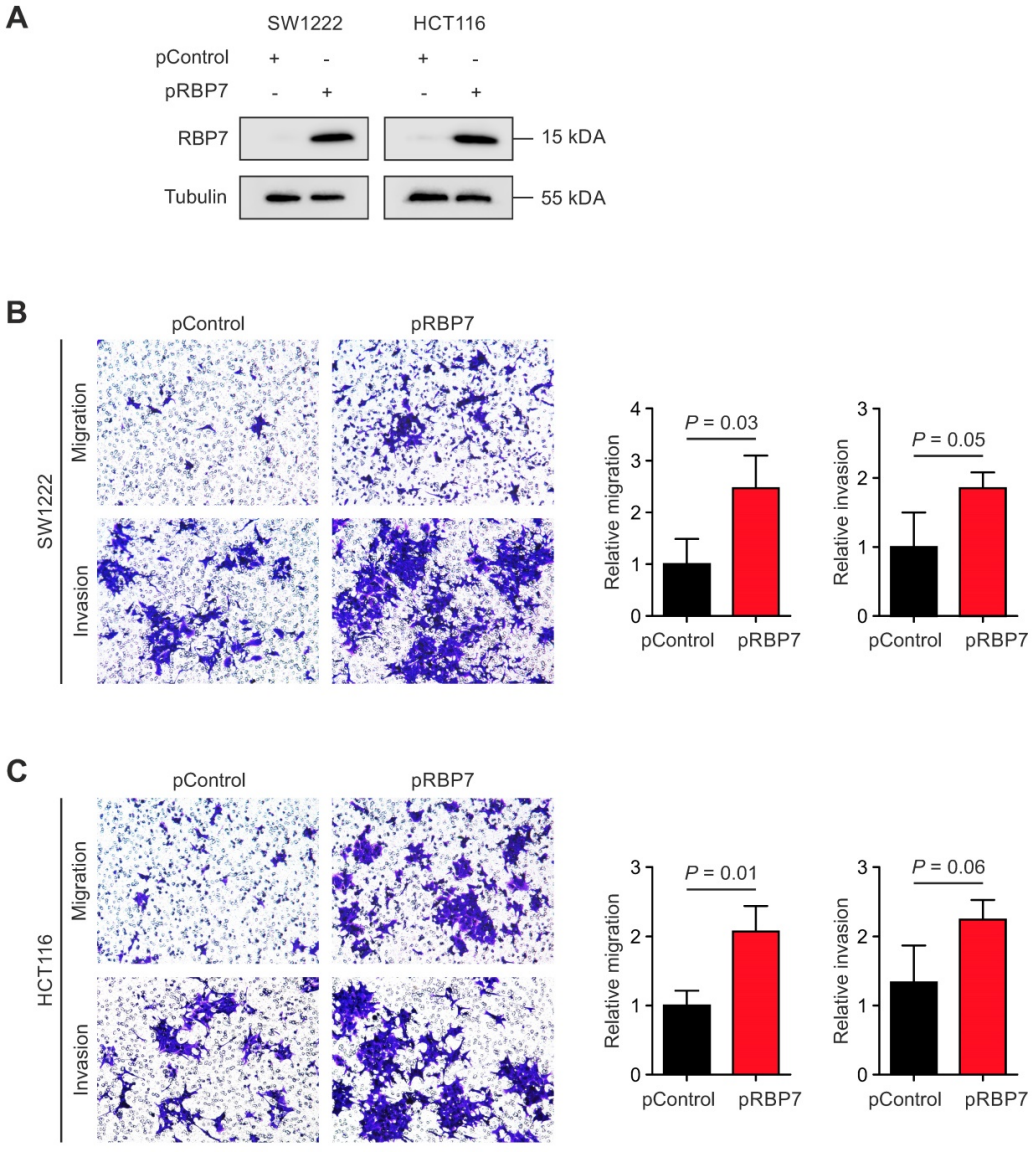
** Overexpression of RBP7 enhances migration and invasion of colon cancer cells**. **(A)** Immunoblotting for indicated proteins on whole cell lysates of SW1222 and HCT116 colon cancer cells harvested 48 h after transfection with pcDNA3.1-RBP7 (pRBP7) or empty pcDNA3.1 (pControl) vector. **(B-C)** Representative micrographs (*left panels*) and quantification (*right panels*) of migrated or invaded **(B)** SW1222 and **(C)** HCT116 colon cancer cells in transwell assays. Data are mean ± SD, n ≥ 3, *P* values are t-test results.

Finally, due to its link with EMT and cancer invasion, we tested for a functional relevance of RBP7 for invasion and migration of colon cancer cells. We constructed a vector for transient overexpression of RBP7. Transfection of HCT116 and SW1222 colon cancer cells with RBP7 encoding vector caused strong ectopic expression in both cell lines, when compared to empty control vector (Figure 4A). We then seeded both cell lines in Boyden chamber assays that were coated with matrigel for invasion. Importantly, ectopic expression of RBP7 increased the number of migrated and invaded tumor cells, and these effects were comparable in both cell lines (Figure 4B-C). These findings supported the idea that RBP7 is a regulator of invasion and migration, which are malignant traits of colon cancer progression.

## Discussion

Here, we demonstrate that RBP7 is a prognostic biomarker in colon cancer. Using a collection of 219 stage I and II colon cancer cases with long-term follow-up data, we show that high levels of RBP7 protein expression were strongly linked to poor cancer specific survival. This finding is important when considering that the clinical management of patients with colorectal cancer is mainly guided by disease stage. While patients with early and localized stage I and II disease generally have the best prognosis, and in most cases can be curatively treated by surgical resection alone, patients with advanced and metastatic disease may benefit from primary or adjuvant chemotherapy 3. However, in about 25-30% of early stage colon cancer cases, the disease still recurs and progresses after surgical management, and may ultimately be fatal 4. Therefore, high expression of RBP7 may be particularly useful to identify patients with colon cancer that are at high risk for disease progression, and thus may be candidates for adjuvant chemotherapy and increased clinical attention, despite low clinical stage. Additionally, we found that also on the mRNA level, *RBP7* was significantly linked with poor outcome in an independent collection of 457 colon cancers from TCGA that included all tumor stages, and also was an independent prognostic biomarker in this case collection. This further validated and broadened the results from our own tissue collection. In addition, in this data set *RBP7* levels increased with T-category and thus with the depth of bowel wall infiltration, suggesting a link of *RBP7* and tumor invasion. Collectively, these findings demonstrated significant biomarker potential of RBP7 on protein and mRNA levels that may be useful for risk stratification in patients with colon cancer.

Disease progression of colon cancer requires invasive growth of tumor cells into surrounding tissues, blood vessels, and lymphatics 22. Invasion often is tied to a loss of epithelial characteristics during epithelial-mesenchymal transition (EMT) 23. Looking at thousands of different curated and hallmark gene sets with GSEA, we found that *RBP7* was most strongly linked to a multicancer invasiveness signature, as well as to a hallmark EMT gene signature 19-21. This is of particular interest because markers that indicate invasion and EMT in colon cancer are scarce, and detection of typical EMT biomarkers such as ZEB1, SNAIL1, or Vimentin in cancer tissues can be challenging, as reflected by yet few convincing *in situ* studies 24, 25. On the contrary, we demonstrate that RBP7 can be robustly detected by immunohistochemistry in primary colon cancer, and quantified by digital image analysis. We therefore suggest that RBP7 may serve as a surrogate marker that indicates the overall degree of tumor invasion and EMT within colon cancer, which also may explain its association with poor prognosis. However, before implementation in a clinical setting, i.e. to complement tumor staging, further independent confirmation of these findings will be mandatory.

Ectopic expression of RBP7 increased migration and invasion, which demonstrates a direct functional contribution of RBP7 to the malignant traits of colon cancer cells. This may explain the association of RBP7 with invasion, EMT, and poor prognosis that we observed in colon cancer case collections. RBP7 belongs to a family of cytoplasmic retinol binding proteins (CRBPs) with important functions in vitamin A uptake, storage and metabolism 9, 10. Previous studies demonstrated that another member, RBP4 and its receptor, are potent oncogenes in human breast and colon cancer cells that drive malignant transformation 12. Furthermore, RBP4 expression in colon cancer has been associated with poor prognosis, promoted growth in xenograft models, and increased the expression of putative cancer stem cell antigens 13. Considering these findings, our data provide a new link of retinol metabolism, invasion, and EMT in colon cancer through RBP7. However, the exact mechanism by which RBP7 drives these malignant traits and affects the transcriptome of colon cancer cells remains to be determined. Further study also is required to elaborate whether therapeutic interference with retinol metabolism and RBP7 may be a strategy to target invasion, EMT, and colon cancer progression.

## Figures and Tables

**Table 1:** Clinical data and RBP7 protein expression in UICC stage I and II colon cancer.

Characteristics	Total	RBP7				*P*
		Low		High		
All patients	219 (100.0)	103 (47.0)		116 (53.0)		
Age (y, Median 69)						
≤ 68	108 (49.3)	51 (47.2)		57 (52.8)		0.956
≥ 69	111 (50.7)	52 (46.8)		59 (53.2)		
Gender						
Male	121 (55.3)	59 (48.8)		62 (51.2)		0.569
Female	98 (44.7)	44 (44.9)		54 (55.1)		
T-category						
T1	1 (0.5)	0 (0.0)		1 (100.0)		0.467
T2	35 (16.0)	17(48.6)		18 (51.4)		
T3	175 (79.9)	84 (48.0)		91 (52.0)		
T4	8 (3.7)	2 (25.0)		6 (75.0)		
UICC-stage						
I	36 (16.4)	17 (47.2)		19 (52.8)		0.989
II	183 (83.6)	86(47.0)		97 (53.0)		
Tumor grade (WHO)						
low grade	197 (90.0)	97 (49.2)		100 (50.8)		0.05
high grade	22 (10.0)	6 (27.3)		16 (72.7)		

Values in parentheses indicate column and row percentage for total and RBP7 low or high cases, respectively.

**Table 2:** Multivariate analysis of cancer specific survival in UICC stage I and II colon cancer.

Variables	Cancer specific survival		
	HR	(95% confidence interval)	*P*
Age ≥ median (69 y)	2.04	(1.06-3.93)	0.032
Female vs. male	0.76	(0.40-1.46)	0.408
T-category	3.32	(1.43-7.70)	0.005
High tumor grade	1.20	(0.52-2.78)	0.669
RBP7 high	2.54	(1.26-5.10)	0.009

**Table 3:** Clinical data and *RBP7* mRNA expression in colon cancer cases from TCGA

Characteristics	Total	*RBP7*				*P*
		Low		High		
All patients	457 (100.0)	332 (72.6)		125 (27.4)		
Age (y, Median 68)						
≤ 67	211 (46.9)	155 (73.5)		56 (26.5)		0.875
≥ 68	239 (53.1)	174 (72.8)		65 (27.2)		
Gender						
Male	235 (52.2)	171 (72.8)		64 (27.2)		0.201
Female	215 (47.8)	158 (73.5)		57 (26.5)		
T-category						
T1	11 (2.5)	11 (100.0)		0 (0.0)		0.00002
T2	77 (16.8)	67 (87.0)		10 (13.0)		
T3	306 (67.0)	222 (72.5)		84 (27.5)		
T4	53 (11.5)	27 (50.9)		26 (49.1)		
Nodal Metastasis						
Negative	263 (58.8)	206 (78.3)		57 (21.7)		0.003
Positive	184 (41.2)	121 (65.8)		63 (34.2)		
Distant Metastasis						
Negative	326 (83.8)	249 (76.4)		77 (23.6)		0.104
Positive	63 (16.2)	42 (66.7)		21 (33.3)		
MSI status						
MSS/MSI-low	340 (81.3)	248 (72.9)		92 (27.1)		0.838
MSI-high	78 (18.7)	56 (71.8)		22 (28.2)		
RAS status						
Mutated	174 (38.1)	130 (74.7)		44 (25.3)		0.691
Wild type	216 (47.3)	153 (70.8)		63 (29.2)		
Unknown	67 (14.7)	49 (73.1)		18 (26.9)		
BRAF (V600E)						
Mutated	47 (10.3)	35 (74.5)		12 (25.5)		0.948
Wild type	343 (75.1)	248 (72.3)		95 (27.7)		
Unknown	67 (14.7)	49 (73.1)		18 (26.9)		

Values in parentheses indicate column and row percentage for total and *RBP7* low or high cases, respectively.

**Table 4:** Multivariate analysis of cancer specific survival in colon cancer cases from TCGA.

Variables	Cancer specific survival		
	HR	(95% confidence interval)	*P*
Age ≥ median (68 y)	0.65	(0.28-1.50)	0.314
Female vs. Male	1.09	(1.09-2.54)	0.847
T-category	2.06	(0.82-5.18)	0.124
Nodal metastasis	2.01	(0.56-7.22)	0.286
Distant metastasis	15.85	(4.66-53.91)	0.00001
MSI-high	2.00	(0.36-10.86)	0.439
*RBP7* high	2.50	(1.05-5.88)	0.038
